# Effect of Plasmonic Gold Nanoprisms on Biofilm Formation and Heat Shock Proteins Expression in Human Pathogenic Bacteria

**DOI:** 10.3390/ph14121335

**Published:** 2021-12-20

**Authors:** Rihab Lagha, Fethi Ben Abdallah, Amine Mezni, Othman M. Alzahrani

**Affiliations:** 1 Department of Biology, College of Sciences, Taif University, P.O. Box 11099, Taif 21944, Saudi Arabia; rihab.k@tu.edu.sa (R.L.); o.alzahrani@tu.edu.sa (O.M.A.); 2 Research Unit UR17ES30: Virology and Antiviral Strategies, Higher Institute of Biotechnology, University of Monastir, Monastir 5000, Tunisia; 3 Department of Chemistry, College of Sciences, Taif University, P.O. Box 11099, Taif 21944, Saudi Arabia; aminemezni@tu.edu.sa

**Keywords:** plasmonic gold nanoprisms, antibacterial, antibiofilm, GroEL/GroES expression, pathogenic bacteria

## Abstract

Gold nanoparticles have gained interest in biomedical sciences in the areas of nano-diagnostics, bio-labeling, drug delivery, and bacterial infection. In this study, we examined, for the first time, the antibacterial and antibiofilm properties of plasmonic gold nanoprisms against human pathogenic bacteria using MIC and crystal violet. In addition, the expression level of GroEL/GroES heat shock proteins was also investigated by western blot. Gold nanoparticles were characterized by TEM and EDX, which showed equilateral triangular prisms with an average edge length of 150 nm. Antibacterial activity testing showed a great effect of AuNPs against pathogenic bacteria with MICs values ranging from 50 μg/mL to 100 μg/mL. Nanoparticles demonstrated strong biofilm inhibition action with a percentage of inhibition ranging from 40.44 to 82.43%. Western blot analysis revealed that GroEL was an AuNPs-inducible protein with an increase of up to 66.04%, but GroES was down-regulated with a reduction of up to 46.81%. Accordingly, plasmonic gold nanoprisms, could be a good candidate for antibiotics substitution in order to treat bacterial infections.

## 1. Introduction

Pathogenic bacteria are microorganisms that cause infectious diseases. They can transmit from person to person directly or indirectly through animal and insect vectors, as well as through polluted water and food. [1]. Antibiotic therapy continues to lose its effectiveness due to the spread of drug resistance in bacterial pathogens. The infections caused by drug-resistant bacteria result in additional very expensive medical costs [2,3]. Faced with this critical situation, the development of new antibacterial agents and therapeutic strategies is urgently necessary.

Most pathogenic bacteria are resistant to antibacterial compounds due to their capacity to produce biofilm [4]. In addition, the biofilm produced by sessile bacteria is responsible for several chronic diseases [5]. Biofilms formed by pathogenic bacteria are one of their major virulence factors that contribute to >80% of human infections [6]. Biofilm is a complex community composed of nucleic acids, lipids, proteins, and polysaccharides [7]. In biofilms, pathogenic bacteria become more persistent in the host environment and more resistant to antibiotics [8,9] that is a major medical problem. Thereby, the discovering of new compounds to combat biofilm is of great importance.

Heat shock proteins (HSPs) are major proteins that may be involved in bacterial biofilm [10]. These HSPs are implicated in the bacterial response to environmental stresses and protect pathogens against phagocytic cells [11]. In bacteria, one of the major molecular chaperones is the GroE machine (GroES and GroEL) [12]. GroEL assists in correct folding and assembly of proteins and is involved in diverse cellular processes, including DNA replication, UV mutagenesis, bacterial growth, RNA transcription, and flagella synthesis [13]. GroEL, together with GroES, translocate protein across membrane barriers. These HSPs act by preventing protein denaturation and to reactivate partially denatured proteins [14,15,16,17].

Nanoparticles have been used successfully in the delivery of therapeutic agents [18]. Due to their physicochemical properties, nanoparticles become more effective against multidrug-resistant pathogens [19]. AuNPs have gained interest in biomedical sciences due to their low toxicity to human cells, chemical, and biological stability, shape, surface properties, catalytic and antibacterial activities, especially in the areas of nano-diagnostics, bio-labeling, and drug delivery [18]. Safe nanomaterial could be a novel approach to inhibit and/or eradicate biofilm-related bacterial infections. In addition, the penetrating power of nanoparticles plays a major role in their action within the biofilm. In addition, the chance of developing resistance to nanoparticles is lower compared to conventional antibiotics [20].

This work aimed to study the antibacterial and biofilm inhibition properties of plasmonic gold nanoprisms against human pathogenic bacteria. In addition, the expression level of heat shock proteins GroEL and GroES was also investigated. 

## 2. Results

### 2.1. AuNPs Characterization 

**Figure 1:**
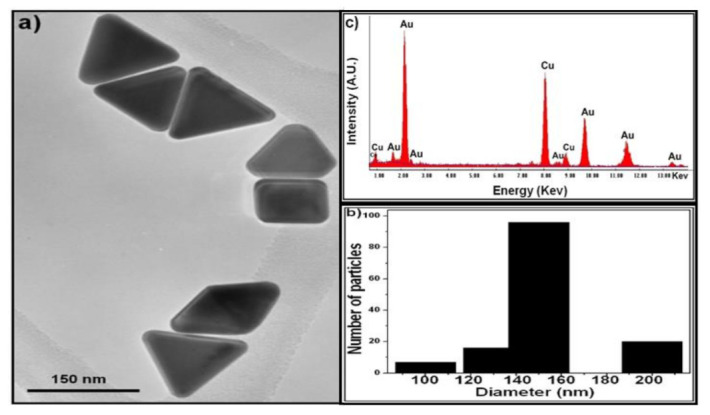
Characterization of AuNPs. (**a**) TEM image, (**b**) particle size distribution, and (**c**) EDX spectrum of triangular gold nanoprisms. Adapted with permission from [21], Copyright 2014 American Chemical Society.

AuNPs were characterized using TEM (Figure 1a) that shows equilateral triangular prisms with an average edge length of 150 nm (Figure 1b). At a molar ratio of PVP/Au = 0.05, the PVP concentration is sufficient to stabilize the gold nanoparticles. Therefore, the gold nanoparticles nucleate more quickly and form monodisperse triangular gold nanoprisms. According to the energy dispersive spectrum (EDX) analysis, le Tr-Au NPs consist of only gold, and the copper element came from a copper grid.

### 2.2. Antibacterial Property of AuNPs

The minimal inhibition concentrations (MICs) and the minimal bactericidal concentrations (MBCs) were used to evaluate the antibacterial activity of AuNPs against the investigated bacteria.

The value of MIC was 50 μg/mL for S. Typhimurium and *V. cholerae*, and was 100 μg/mL for *E. coli*, *E. faecalis*, *P. aeruginosa*, *S. aureus*, *B. cereus*, *S. sonnei*, and *N. gonorrhoeae*. However, the MBC values were 0.4 mg/mL for all the tested bacteria excepted from *S. aureus* and *S. sonnei*, which were 0.8 mg/mL.

### 2.3. Biofilm Formation

Bacteria were evaluated for their ability to produce biofilm on polystyrene surfaces (Table 1). Results showed that almost all of the strains were low-grade positive producers with OD570 values varied from 0.119 to 0.599. In addition, only *S. aureus* was a highly positive producer, while *B. cereus* and *V. cholerae* were not able to form biofilms.

### 2.4. Biofilm Inhibition

Biofilm inhibitory activity of AuNPs was assessed on the strains that showed a biofilm formation potential on polystyrene surface. 

According to Table 1, AuNPs showed great antibiofilm activity. On polystyrene, the percentage of biofilm inhibition ranged from 40.44 to 82.43%. The greatest antibiofilm activity was detected from S. Typhimurium, which passed from low-grade positive to biofilm negative. In addition to *S*. *aureus* that changed from highly positive to and low-grade positive and *P. aeruginosa* that changed from highly positive to biofilm negative. Moreover, the two low-grade positive *E. faecalis* and *N. gonorrhoeae* have conserved their initial biofilm phenotype despite the observed inhibition. However, AuNPs did not show any activity on *E. coli* and *S. sonnei*.

### 2.5. Effect of AuNPs on GroES and GroEL Expression

**Figure 2:**
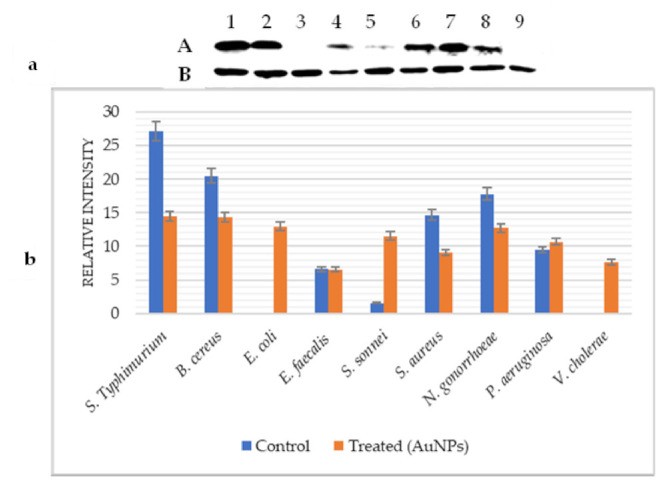
GroES heat shock protein expression under AuNPs effect. (**a**): Western blot analysis; (**b**): Image-J bands quantification; (A): Control; (B): Treated (AuNPs). S. Typhimurium (1), *B. cereus* (2), *E. coli* (3), *E. faecalis* (4), *S. sonnei* (5), *S. aureus* (6), *N. gonorrhoeae* (7), *P. aeruginosa* (8), and *V. cholerae* (9).

The expression level of GroES before and after treatment with AuNPs was analyzed using western blot (Figure 2a). Control cells showed that GroES was expressed in all tested bacteria with different degrees except for *E. coli* and *V. cholerae*. The very high and low expression levels of this protein were observed in *S. typhimurium* and *S. sonnei*, respectively.

Under the AuNPs effect, the analysis by Image-J (Figure 2b) demonstrated that the quantity of GroES protein was reduced in S. Typhimurium, *B. cereus*, *S. aureus*, and *N. gonorrhoeae* with percentages of 46.81%, 30.17%, 38.35%, and 28.43%, respectively. However, the amount of GroES protein was increased in *S. sonnei* (86.33%) and *P. aeruginosa* (11.40%). In addition, this protein remained stable in *E. faecalis* but was expressed in *E. coli* and *V. cholerae*.

**Figure 3:**
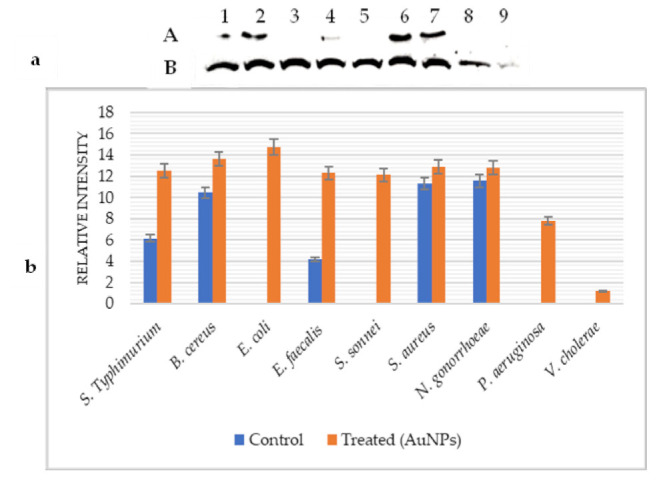
GroEl heat shock protein expression under AuNPs effect. (**a**): Western blot analysis; (**b**): Image-J bands quantification; (A): Control; (B): Treated (AuNPs) S. Typhimurium (1), *B. cereus* (2), *E. coli* (3), *E. faecalis* (4), *S. sonnei* (5), *S. aureus* (6), *N. gonorrhoeae* (7), *P. aeruginosa* (8), and *V. cholerae* (9).

Concerning GroEL (Figure 3a), the results showed that this protein was expressed with different degrees only in the non-treated S. Typhimurium, *B. cereus*, *E. faecalis, S. aureus*, and *N. gonorrhoeae*. 

After treatment with AuNPs, GroEL was induced in all tested bacteria. Its level of expression was considerably increased in *E. faecalis* (66.04%), S. Typhimurium (50.83%), and *B. cereus* (23.35%). This Hsp was slightly induced in *S. aureus* (12.5%) and *N. gonorrhoeae* (9.64%). The very high and low expression levels of GroEL were detected in *E. coli* and *V. cholerae*, respectively (Figure 3b).

## 3. Discussion

The increased resistance of pathogenic bacteria to antibiotics and the lack of new antibacterial drugs have prompted researchers to focus their efforts to explore nanotechnological measures against microbial infections for therapeutic applications. AuNPs have attracted considerable interest because of their promising applications in the development of novel antibacterial molecules [1]. This study investigates for the first time the effect of plasmonic gold nanoprisms on biofilm formation and GroEL/GroES proteins expression in some pathogenic bacteria as well as their antibacterial properties.

The antibacterial activity showed that AuNPs are effective against tested pathogenic bacteria. The MICs values varied from 50 μg/mL, for *S. typhimurium* and *V. cholerae*, to 100 μg/mL for *E. coli*, *E. faecalis*, *P. aeruginosa*, *S. aureus*, *B. cereus*, *S. sonnei*, and *N. gonorrhoeae*. Accordingly, Gram-negative and Gram-positive bacteria react in the same way face to AuNPs. However, the study of Zawrah et al. [22] indicated that AuNPs are less effective against Gram-positive bacteria due to the nature of the cell wall. Nanoparticles, with a positive charge, attach with the negatively charged microorganisms by the electrostatic attraction in the cell wall membrane [23].

AuNPs characterized using TEM appear as equilateral triangular prisms with an average edge length of 150 nm. Several reports have shown that smaller NPs have higher antibacterial activity [24,25,26]. Indeed, smaller particles affect the larger surface area of the bacteria, enter easily into the bacterial cell and affect the intracellular processes. Thereby they have more bactericidal activity than larger [27]. However, in this work, the triangular prisms of gold showed great antimicrobial property, despite their high length, against Gram-positive and Gram-negative bacteria. This finding corroborates the data of Sohm et al. [28] showing that larger NPs are more effective and indicating that size alone is not the most important factor of their toxicity. The effectiveness of gold triangular nanoprisms may be due to their shape. According to Smitha and Gopchandran [29], the antibacterial properties of triangular AuNPs have shown better activity against Gram-positive and Gram-negative bacteria compared to spherical AuNPs, which indicates that the shape could play a significant role in the potential antibacterial activity of AuNPs.

Biofilm, as a major virulence factor, is responsible for about 80% of human infections, and the Gram-negative bacterium *P. aeruginosa*, *E. coli*, and the Gram-positive *S. aureus* are the most frequent [30]. Due to the resistance to antibiotics, the immune system, and the spread of infection, the treatment of biofilm becomes more complex [31]. Thereby, the discovering of novel therapeutic molecules, like plasmonic gold nanoprisms, to inhibit biofilm is of great importance. In this work, almost all of the tested bacteria were low-grade positive producers on polystyrene surfaces with OD570 values ranging from 0.119 to 0.599. In addition, only *S. aureus* was a highly positive producer, while *B. cereus* and *V. cholerae* were not able to form biofilm. AuNPs showed great antibiofilm activity with the percentage of inhibition ranging from 40.44% for *N. gonorrhoeae* to 82.43% for *S. typhimurium*. Thereby, this nanoparticle could be a good alternative for the treatment of the formation of biofilm by pathogenic bacteria. Indeed, the penetration ability of AuNPs to get inside the biofilm and to disperse is of great importance as the biofilm layer provides an impermeable barrier to many antibiotics. Our results are in agreement with those of Singh et al. [32], who showed that AuNPs possess a remarkable reduction in the biofilm formation by *P. aeruginosa* and *E. coli*. The highest antibiofilm activity of AuNPs was observed in *S. typhimurium* and *S. aureus* that formed the strongest biofilm among the tested bacteria. However, this result is in discordance with the report of Ahmed et al. [33], who observed lower disruption of biofilms in the strong biofilm producer *K. pneumoniae* compared to the other isolates suggesting that the thickness and composition of biofilm play a key role in the penetration of AuNPs. This may be attributed to the shape of AuNPs as equilateral triangular prisms.

Hsps are key elements in the bacterial response to stress and environmental changes in order to maintain cell homeostasis in addition to their important role in pathogenesis [34]. GroEL/GroES play a major role in protein folding even during non-stressed growth conditions, although their action becomes more important during stress. In this work, we investigated the differential expression of GroEL and GroES under the effect of AuNPs. Firstly, before treatment, GroES was expressed with different levels in all tested bacteria except for *E. coli* and *V. cholerae*, but GroEL was expressed only in *S*. *typhimurium*, *B. cereus*, *E. faecalis, S. aureus*, and *N. gonorrhoeae* which indicates that GroES is more implicated in normal growth bacteria than GroEL. Under AuNPs effect, the amount of GroEL was considerably increased in all strains indicating that this protein plays an important role in maintaining the cell in such condition, which is in agreement with the report of Makumire et al. [35]. Thereby it can be considered as an AuNPs-inducible protein [36]. Concerning GroES, its production was increased only in *S. sonnei* and *P. aeruginosa* but was decreased or remained stable in other strains suggesting that this protein was altered or downregulated by AuNPs. According to Kustos et al. [37], under stress conditions such as nanoparticles, protein synthesis is inhibited, and cell division is interrupted. In parallel, the expression of various proteins increases; these are the so-called stress proteins as GroEL in this study. Knowing that the GroE machine is involved in protein folding and other mechanisms, the overexpression of GroEL may have served as compensation for the lack of GroES expression to maintain cell homeostasis. However, alteration observed in GroES expression may indicate that the GroE machine was altered, and thereby the protein folding mechanism of bacteria may be altered, which can explain the high activity of AuNPs.

## 4. Materials and Methods

### 4.1. Bacterial Strains

Nine (9) human pathogenic bacteria were tested in this work included: *S*. *typhimurium* (ATCC 1408), *E. coli* (ATCC 35218), *S. sonnei* (ATCC 25931), *E. faecalis*, *P. aeruginosa* PAO1, *S. aureus* (ATCC 25923), *B. cereus* (ATCC 11778), *V. cholerae* (ATCC 9459), and *N. gonorrhoeae* (ATCC 49226).

### 4.2. Gold Nanoparticles

#### 4.2.1. Synthesis

To synthesize gold nanoparticles, a triethylene glycol reagent (ACROS Organics, 98%) was used as a solvent. The precursor of gold was hydrogen tetrachloroaurate (III) trihydrate (HAuCl_4_·3H_2_O; Sigma-Aldrich, St. Louis, MO, USA). In addition, polyvinyl-pyrrolidone (PVP) (K30, Sigma–Aldrich) was used as a surfactant [21]. For the experiment, 25 mL of triethylene glycol suspension containing 0.038 mmol of HAuCl_4_·3H_2_O and a given quantity of PVP were heated at 150 °C for 30 min under shaking. The molar ratio of PVP to HAuCl_4_ (R(PVP/Au)) was 0.05. The formed gold particles and the final colloidal solution had a blue color. The product was centrifuged, washed many times with ethanol/acetone (2:1) solution, and scattered in ethanol.

#### 4.2.2. Characterization

The morphological analysis of the gold particles was determined by transmission electron microscopy (TEM) (JEOL-JFC 1600). An elemental analysis was conducted using energy-dispersive X-ray spectrograph (EDX) attached to the TEM. The selected area electron diffraction (SAED) was also performed on the microscope. The Perkin-Elmer Lambda 11 UV/VIS spectrophotometer was used to determine the optical absorption spectra of diluted Au NPs solution.

### 4.3. Antibacterial Activity of Au NPs: Minimum Inhibitory and Minimum Bactericidal Concentration

MICs and MBCs were determined three times on 96-well microtiter plates (Nunc, Roskilde, Denmark) [38]. The bacterial inoculums (0.5 McFarland standards turbidity) were prepared from 12 h broth cultures. Then, a serial two-fold dilution of the AuNPs was prepared in 5 mL of nutrient broth with a concentration ranging from 0.012 to 7.2 mg/mL.

The plates were prepared by adding 100 μL from the AuNPs serial dilutions, 95 μL of nutrient broth, and 5 μL of the bacterial inoculum in each well. The negative control comprising 5 μL of the inoculum and 195 μL of nutrient broth without AuNPs was placed in the last well. The plates at a final volume of 200 μL were then incubated at 37 °C for 18–24 h. 

The lowest concentration of the AuNPs at which the growth of the cells was inhibited was interpreted as MIC. However, the lowest concentration of AuNPs, required to kill ≥99.9% of the initial bacterial cells was interpreted as MBC and was determined by subculturing 20 μL from clear wells of the MIC test on MHA [39].

### 4.4. Biofilm Formation on Polystyrene

The capacity of bacteria to form biofilm was evaluated by crystal violet assay using U-bottomed 96-well microtiter plates [40]. Each bacterium was investigated three times, and sterile TSB was served as control. An automated Multiskan reader (GIO. DE VITA E C, Rome, Italy) was used to measure the wells’ optical density at 570 nm (OD570). Biofilm formation was defined as highly positive (OD570 ≥ 1), low-grade positive (0.1 ≤ OD570 < 1), or negative (OD570 < 0.1).

### 4.5. Biofilm Inhibition

The capacity of AuNPs to prevent bacterial strains from forming biofilms was investigated. Experimentally, 100 µL of AuNPs mixed in TSB (2% glucose) were placed in each well of a U-bottomed 96-well microtiter plate, along with 100 µL of bacterial suspensions (10^8^ CFU/mL). The final AuNPs concentrations were equivalent to the MIC, and the final volume per well was 200 µL. The experiments were carried out three times. Crystal violet was used to measure the formed biofilm after incubation of microplates at 37 °C for 24 h [41]. The inoculum volume and AuNPs were replaced with TSB and sterile water in the control wells, respectively. The percentage of biofilm inhibition was determined according to the formula described by Jadhav et al. [42].

(1) $$\%\;\mathrm{Inhibition}\;=\;100\;-\;(\frac{\mathrm{OD}570\;\mathrm{sample}}{\mathrm{OD}570\;\mathrm{control}}\;\times \;100)$$

### 4.6. Western Blot for GroEL and GroES Analysis

Expression of GroEL and GroES under AuNPs conditions were analyzed using Western blot. For experiments, 11µg of whole-cell proteins (control and AuNPs treated bacteria) were analyzed by SDS-PAGE [43] and blotted onto nitrocellulose membranes (Millipore, Bedford, MA, USA). The membranes were blocked overnight at 4 °C in PBS-5% skimmed milk, 0.1% Tween 20. The membranes were incubated for 1 h with anti-GroEL (Abcam, ab90522) and anti-GroES (Abcam, ab69823) polyclonal rabbit-antibodies were diluted to 1/1000 and 1/5000, respectively. Then, the membranes were incubated with a goat anti-rabbit IgG peroxidase-conjugated monoclonal antibody (Sigma, St Louis, MO, USA) diluted at 1/10,000. The bound antibodies were visualized by ECL (GE Healthcare, Uppsala, Sweden). Western blots were analyzed with imaging (Image-J 1.50) to determine the intensity of each band [44].

## 5. Conclusions

The results developed in this work support the medical application of AuNPs as a therapeutic molecule for the treatment of infections caused by pathogenic bacteria. Plasmonic gold nanoprisms showed, for the first time, great antibacterial and antibiofilm activities against human pathogenic bacteria. In addition to the alteration observed in GroES expression as a part of GroE machine implicated in the bacterial protein folding mechanism. Despite their stability and low toxicity, the application of AuNPs needs more in vitro testing and in vivo clinical trials to establish their safety, efficacity, and possible adverse effects.

## Figures and Tables

**Table 1:** Antibiofilm activity of AuNPs on polystyrene.

Strains	Control OD570 ± SD	Biofilm Phenotype	AuNPs OD570 ± SD	Biofilm Phenotype	Inhibition (%)
*S. Typhimurium*	0.37 ± 0.026	low-grade positive	0.065 ± 0.073	negative	82.43
*B. cereus*	0.046 ± 0.003	negative	-	-	-
*E. coli*	0.119 ± 0.042	low-grade positive	0.12 ± 0.062	low-grade positive	0
*E. faecalis*	0.599 ± 0.067	low-grade positive	0.206 ± 0.032	low-grade positive	65.60
*S. sonnei*	0.142 ± 0.022	low-grade positive	0.131 ± 0.046	low-grade positive	0
*S. aureus*	3.22 ± 0.088	highly positive	0.6 ± 0.058	low-grade positive	81.36
*N. gonorrhoeae*	0.361 ± 0.014	low-grade positive	0.215 ± 0.023	low-grade positive	40.44
*P. aeruginosa*	0.409 ± 0.076	low-grade positive	0.084 ± 0.016	negative	79.46
*V. cholerae*	0.032 ± 0.002	negative	-	-	-

## Data Availability

Data is contained within the article.
